# Investigating the Determinants of *Toxoplasma gondii* Prevalence in Meat: A Systematic Review and Meta-Regression

**DOI:** 10.1371/journal.pone.0153856

**Published:** 2016-04-15

**Authors:** Simone Belluco, Marzia Mancin, Daniele Conficoni, Giulia Simonato, Mario Pietrobelli, Antonia Ricci

**Affiliations:** 1 Food Safety Department, Istituto Zooprofilattico Sperimentale delle Venezie, viale dell’Università 10, 35020, Legnaro, PD, Italy; 2 Department of Animal Medicine, Production and Health, University of Padua, Viale dell’Università, 35020, Legnaro, PD, Italy; Auburn University, UNITED STATES

## Abstract

**Background:**

*Toxoplasma gondii* is one of the most widespread parasites in humans and can cause severe illness in immunocompromised individuals. However, its role in healthy people is probably under-appreciated. The complex epidemiology of this protozoan recognizes several infection routes but consumption of contaminated food is likely to be the predominant one. Among food, consumption of raw and undercooked meat is a relevant route of transmission, but the role of different meat producing animal species and meats thereof is controversial.

**Objectives:**

The aim of the present work is to summarize and analyse literature data reporting prevalence estimates of *T*. *gondii* in meat animals/meats.

**Data Sources:**

We searched Medline, Web of Science, Science Direct (last update 31/03/2015).

**Eligibility Criteria:**

Relevant papers should report data from primary studies dealing with the prevalence of *T*. *gondii* in meat from livestock species as obtained through direct detection methods. Meta-analysis and meta-regression were performed.

**Results:**

Of 1915 papers screened, 69 papers were included, dealing mainly with cattle, pigs and sheep. Pooled prevalences, based on random-effect models, were 2.6% (CI_95_ [0.5–5.8]) for cattle, 12.3% (CI_95_ [7.6–17.8]) for pigs and 14.7% (CI_95_ [8.9–21.5]) for sheep. Due to the high heterogeneity observed, univariable and multivariable meta-regression models were fitted showing that the geographic area for cattle (p = 0.032), the farming type for pigs (p = 0.0004) and the sample composition for sheep (p = 0.03) had significant effects on the prevalences of *Toxoplasma* detected/estimated. Moreover, the role of different animal species was dependent on the geographic location of animals’ origin.

**Limitations:**

Limitations were due mainly to a possible publication bias.

**Conclusions and Implications:**

The present work confirms the role of meat, including beef, as *T*. *gondii* sources, and highlights the need for a control system for this parasite to be implemented along the meat production chain. Moreover, consumer knowledge should be strengthened in order to reduce the impact of disease.

## Introduction

Toxoplasmosis is a zoonotic disease caused by *Toxoplasma gondii*, one of the most widespread parasites among humans. The clinical importance of this disease is due largely to infection occurring during pregnancy or in immunocompromised individuals [1]. In contrast, its impact on healthy individuals is probably underestimated. Toxoplasmosis can cause serious health problems in immunocompetent people [1–4], and the parasite can reactivate in chronically infected individuals as a consequence of immunosupression due, for example, to organ transplant or HIV infection. In addition, there is a growing interest in the study of the potential relationship between *T*. *gondii* latent infections and neurological disorders [5]. *T*. *gondii*, both congenital and perinatal, has the greatest impact on public health in terms of Disability Adjusted Life Years (DALY) among all foodborne pathogens according to a study performed in the Netherlands [6], and the burden is suggested to be even higher in other countries [7].

The complex life cycle of *T*. *gondii* recognizes felids as definitive hosts, in which the parasite can complete its sexual cycle and from there spread millions of oocysts into the environment. Although the number of oocysts produced is a key element in environmental contamination and consequently in parasite transmission, *T*. *gondii* is able to rely also on its asexual cycle in almost all warm blooded animals. This is a key adaptation of life cycle [8], and enables the parasite to be transmitted through the ingestion of infected meat, as observed several decades ago [9]. Consumption of raw or undercooked meat is likely to be the major transmission route for humans [10].

*T*. *gondii* infection in food producing animals is a critical issue and, despite the high number of studies estimating prevalence through serology and/or direct detection of the parasite in animal samples, there is disagreement about the relative importance of different food animal species. The most controversial role concerns cattle. Their importance in *T*. *gondii* transmission was judged to be unresolved several years ago as the parasite was never isolated from beef tissue [11]. Moreover, a large study performed in the US recently failed to detect *T*. *gondii* in more than 2000 samples, supporting the theory that cattle are a poor host for the parasite [12]. In contrast, other authors support different theories. For example, Opsteegh and colleagues, despite agreeing on the low prevalence in cattle, argued that the risk posed to consumers by ingestion of contaminated beef is likely to be high due to consumption habits [13]. Efforts to collect data on *T*. *gondii* prevalence have been made [14,15] but without recourse to meta-analysis, which, together with meta-regression, is a helpful technique to obtain insight into the reasons for such differences and to depict the current knowledge in an evidence-based way.

The aims of the present study are to systematically review literature on the prevalence and determinants of *T*. *gondii* in meat of food producing animals and analyse the data through meta-analysis and meta-regression.

## Methods

### Data sources and searches

Relevant studies were identified by searching multiple literature databases including Medline (through PubMed), Web of Science Core Collection, SciELO citation index (through Web of Science) and Science Direct. No time limitation was imposed. The search was executed on 30/06/2014 and last updated on 31/03/2015.

The search string used was the following: (Toxoplasma OR Toxoplasmosis) AND (“Dairy Products” OR Meat OR poultry OR beef OR pork OR horse OR vegetables OR milk OR consumption OR food OR carcas*). Only papers in English, Italian, French, Spanish and Portuguese were considered. References were imported in EPPI-4 software [16] and duplicates were removed. Relevant papers were manually cross checked in order to identify further references.

### Study selection and data extraction

Several criteria were used to select eligible studies: 1) the prevalence of *T*. *gondii* had to be detected by direct methods (bioassay, PCR, microscopy); 2) samples had to originate from food of animal origins (except milk and dairy products) belonging to the main livestock species (cattle, pigs, sheep, goat and horses); 3) samples had to be collected from animals which had not been experimentally infected; 4) sampling strategy had to be directed toward a random population.

**Fig 1:**
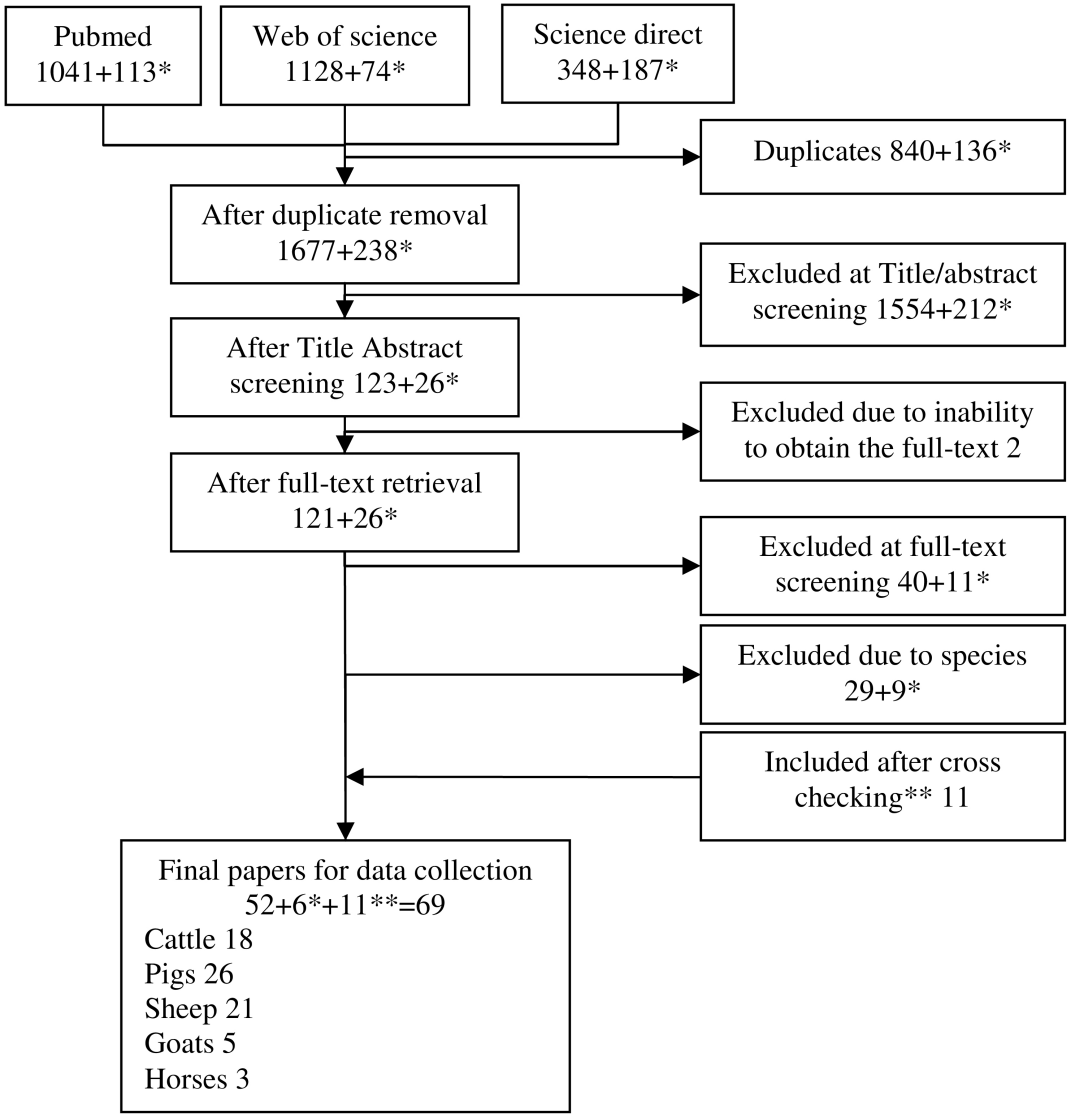
Flowchart describing the selection of relevant studies. *papers added during the last update ** papers added through cross checking.

The selection process is detailed in Fig 1. Briefly, the screening process, both Title/Abstract and Full text, was performed by two reviewers (SB, DC) independently (parallel method). Disagreements were resolved through consensus. Data were extracted by one reviewer and checked by a second (sequential method). All studies were coded according to the previously chosen parameters and data were recorded on customized tables. The collective noun for each animal species (cattle, pigs, sheep, horses and goats) is used throughout the current paper to describe tissue (mostly edible) deriving from that meat-producing animal species.

### Risk of bias in individual studies

Study-level risk of bias was likely to be high mainly because of differences in study design and sampling management. Studies describing a sampling campaign on farms already recognized as being at risk were excluded [17,18]. Additional efforts were made to collect data about randomization and sample selection, such as size of the population from which the animals originated, method of selection of individuals and geographic distribution, but these factors were poorly described in primary studies, impairing further analysis.

The minimum sample size was set to ten, and this choice caused the exclusion of two studies reporting data for cattle and pigs with four and nine samples respectively [19]. In addition, the impact of sample size on the pooled prevalence estimate was assessed, for each species, through a cumulative meta-analysis based on decreasing sample size. Moreover, potential sources of bias, such as sample composition, analytical technique and study design were assessed through meta-regression. Outcome-level biases were not evaluated. However, an accurate sensitivity analysis was performed to detect influential studies.

The outcome selected for meta-analysis (event rate, defined as the number of events over the total sample size) was obtained from studies with the following rules. If studies reported different prevalence estimates obtained through different analytical methods or in different target organs, the highest value was retained (prevalence at animal level), assuming that it represented the most sensitive estimate. When direct methods were applied only to seropositive samples, the proportion of positives was adjusted according to the size of the entire study population (i.e. 100 animals in the population, 50 seropositive, 10% of seropositive confirmed through direct method, prevalence in total population = 5%). Moreover, when direct methods were applied only to a fraction of seropositive animals, the proportion of positives was adjusted pro rata considering all seropositives and then by calculating according to the size of the original population (i.e. 100 animals in the population, 50 seropositive animals, 30 seropositive animals tested through direct method, 10 confirmed positive, prevalence in total population = ((30*10)/50)*100 = 16.7%).

### Data analysis

#### Pooled prevalence

Meta-analyses were performed using the *metafor* package [20] of the statistical software R [21]. The proportion of positives among the total study population (event rate) was chosen as effect size. A study was designated as the unit of analysis, and was defined as an investigation performed on a group of animals which shared the same features (e.g. species, geographic location) in terms of variables used as moderators.

Meta-analysis is a statistical method that combines outcomes of primary studies with a weight assigned according to the inverse of the variance. For this reason, the variance is a critical parameter to be taken into account, and must also be calculated when studies reporting zero prevalences are included. The Freeman and TukeyDoubleArcsin transformation of the prevalence was used to obtain a variance stabilizing transformation without applying continuity corrections or removing studies from the meta-analysis, and to give an appropriate weight to those studies with zero prevalence and high numerousness [20,22]. Transformed prevalence estimates were combined in meta-analysis using a random-effect model and later back-transformed in the original metrics. The amount of heterogeneity was estimated using the Q, T^2^ and I^2^ [23] statistics obtained by Restricted Maximum Likelihood (REML), which is considered approximately unbiased and relatively efficient [24]. A separate meta-analysis was performed for each species (cattle, pigs and sheep). Data belonging to goats and horses were only described qualitatively.

Sensitivity analyses were performed in order to evaluate the presence of outliers or leverage studies and their potential influence on each model per species. Several parameters were examined: the externally studentized residuals, the DFFITS (DiFference in FIT, Standardized), the Cook’s distance, the hat function and the covariance ratio. Influence was defined according to *metafor* package criteria (absolute DFFITS value > 3√[p/(k-p)], where p is the number of model coefficients and k is the number of studies OR the lower tail area of a chi-square distribution with p degrees of freedom cut off by the Cook’s distance being larger than 50% OR hat value > 3(p/k)). In addition, studies were excluded one by one from the model to evaluate relevant changes in heterogeneity (T^2^ and Q) and pooled estimate. P-value<0.05 was considered significant in the statistical meta-analysis.

#### Heterogeneity

Heterogeneity was explored through uni-variable and multivariable meta-regression using the mixed-effects models [25]. Moderator significance for (nested) models was assessed through the Likelihood Ratio Test (LRT) by comparing the proportional reduction in the amount of heterogeneity (T^2^ value) of the full and reduced models. Therefore, it was possible to evaluate the amount of (residual) heterogeneity accounted for by the moderator (R^2^). Maximum Likelihood (ML) estimate instead of REML was use to evaluate the importance of the moderators [20].

Attempts were made to explain heterogeneity through epidemiological and methodological moderators: publication year (as a proxy variable for study year), geographic origin, animal age, farming system, analytical technique, sampling location, serological screening presence and sample composition (details in Table 1). In addition, a multivariable meta-regression was performed, pooling all studies across species. This allowed us to use species as moderator and to evaluate the interaction between species and geographic area. In cases of moderator significance, determined according to the Likelihood Ratio Test, a pairwise comparison multitest was performed using the False Discovery Rate correction [26]. Publication bias was evaluated through the Trim and Fill method [27,28] and cumulative meta-analysis was based on sample size.

**Table 1:** Characteristics of 91 studies reporting prevalence for *T*. *gondii gondii* that were tested as sources of heterogeneity.

	Cattle		Pigs		Sheep	
	K	N	K	N	K	N
**Total**	21	3785	41	10894	29	4150
**EPIDEMIOLOGICAL MODERATORS**						
*Geographic area*						
-Africa	-	-	2	100	4	351
-Asia	3	170	2	439	8	376
-Central America	3	100	1	48	-	-
-Europe	4	651	14	3074	8	1061
-North America	3	2429	7	6318	2	469
-Oceania	1	80	1	30	2	64
-South America	7	355	14	885	5	1829
*Animal age*						
-<12 months	-	-	-	-	8	991
->12 months	-	-	-	-	9	543
-NS	-	-	-	-	12	2616
*Farming system*						
-Conventional	-	-	2	397	-	-
-Organic	-	-	4	86	-	-
-Small farms	-	-	2	433	-	-
-NS	-	-	33	9978	-	-
*Publication Year*						
**METHODOLOGICAL MODERATORS**						
*Analytical technique*						
-Bioassay in cats	2	2369	1	2094	-	-
-Bioassay in mice	12	600	28	5695	11	2137
-PCR	7	816	12	3105	16	1913
-Microscopy	-	-	-	-	2	100
*Sample type*						
-Single	14	3095	21	4670	17	2123
-Pooled within animal	7	690	12	5321	12	2027
-Meat products	-	-	5	336	-	-
-Cured meat products	-	-	3	567	-	-
*Sampling location*						
-Slaughterhouse	9	1315	21	7094	21	3686
-Retail	9	2332	18	3739	7	414
-NS	3	138	2	61	1	50
*Serological screening*						
-No	-	-	35	6808	22	1946
-Yes	-	-	6	4086	7	2204

K = number of studies (note that some individual published papers contained more than one study), N = number of samples, NS = non specified in the primary study.

## Results

### Study selection and data extraction

The original literature search provided a total of 1677 records after duplicate removal, and 238 records were obtained during the last update (Details in Fig 1). After the first screening based on Title and Abstract, 149 papers remained and at the end of the selection procedure, 69 of which were considered as relevant according to the eligibility criteria. The final number included papers belonging to the original literature search, papers belonging to the last update and papers retrieved through cross checking the references in the included papers. Studies were identified within the included papers and coded according to review criteria. Details showing the result of study coding on the basis of relevant characteristics are presented in Table 1, whereas details for each eligible study are shown in Tables 2–4.

**Table 2:** General information about eligible studies reporting data for cattle.

ID	Reference	Country	Geographic area	Sampling location	Analytical technique	Technique specifications	Sampled organ
1	**Arias 1994**	Costa Rica	Central America	Retail	Bio mice	Feed	Liver
2	**Arias 1994**	Costa Rica	Central America	Retail	Bio mice	Feed	Heart
3	**Arias 1994**	Costa Rica	Central America	Retail	Bio mice	Feed	Muscle
4	**Azizi 2014**	Iran	Asia	NS	PCR	Nested	Brain/Liver/Muscle
5	**Berger Scoch 2011**	Switzerland	Europe	Slaughterhouse	PCR	Real-T PCR	Diaphragm
6	**Campo-Portacio 2014**	Colombia	South America	Retail	PCR	Nested	Muscle
7	**Catar 1969**	Czech Republic	Europe	NS	Bio mice	IP	Brain/Diaphragm
8	**Dubey 1976**	US	North America	Slaughterhouse	Bio cats	Feed	Heart/Diaphragm
9	**Dubey 2005**	US	North America	Retail	Bio cats	Feed	Muscle
10	**Ergin 2009**	Turkey	Asia	Slaughterhouse	PCR	Nested	Brain/Muscle
11	**Fortier 1990**	Portugal	Europe	Slaughterhouse	Bio mice	IP	Brain/Heart/Diaphragm
12	**Jacobs 1960**	US	North America	Slaughterhouse	Bio mice	IP	Diaphragm
13	**Jacobs 1963**	New Zealand	Oceania	Slaughterhouse	Bio mice	IP	Diaphragm
14	**Jamra 1969**	Brazil	South America	Retail	Bio mice	IP	Muscle
15	**Jamra 1969**	Brazil	South America	Retail	Bio mice	IP	Liver
16	**Jamra 1969**	Brazil	South America	Retail	Bio mice	IP	Brain
17	**Martins 1989**	Brazil	South America	NS	Bio mice	IP	Muscle
18	**Opsteegh 2011**	The Netherlands	Europe	Slaughterhouse	PCR	MC-PCR	Heart
19	**Passos 1984**	Brazil	South America	NS	Bio mice	IP	Diaphragm
20	**Rahdar 2012**	Iran	Asia	Slaughterhouse/ Retail	PCR	PCR	Tongue/Heart/Muscle
21	**Santos 2010**	Brazil	South America	Slaughterhouse	PCR	Nested	Brain/Heart

Bio mice = Bioassays in mice, IP = intra-peritoneal, MC-PCR = magnetic capture PCR, NS = not specified in the primary study.

**Table 3:** General information about eligible studies reporting data for pigs.

ID	Reference	Country	Geographic area	Farm	Serological screening	Analytical technique		Sampled organ
1	**Aspinall 2002**	UK	Europe	NS	NA	PCR	PCR	Meat products (Mixed)
2	**Bacci 2015**	Italy	Europe	O	NA	PCR	Nested	Heart
3	**Bayarri 2012**	Spain	Europe	NS	NA	Bio mice	IP	Muscle
4	**Bayarri 2012**	Spain	Europe	NS	NA	Bio mice	IP	Cured Ham
5	**Belfort-Neto 2006**	Brazil	South America	NS	NA	PCR	PCR	Tongue/Diaphragm
6	**Berger Scoch 2011**	Switzerland	Europe	NS	NA	PCR	Real-T PCR	Diaphragm
7	**Bezerra 2012**	Brazil	South America	O	NA	Bio mice	SC	Brain/Tongue
8	**Cademartori 2014**	Brazil	South America	SF	Sero +	Bio mice	IP	Brain/heart
9	**Catar 1969**	Czech Republic	Europe	NS	NA	Bio mice	IP	Brain/Diaphragm
10	**Clementino andrade 2013**	Brazil	South America	NS	Sero +	Bio mice	IP	Heart
11	**Dias 2005**	Brazil	South America	NS	NA	Bio mice	Inoculation	Sausages
12	**Dubey 1995**	US	North America	NS	NA	Bio mice	SC	Heart
13	**Dubey 2005**	US	North America	NS	Bio cats	Bio cats	Feed	Muscle
14	**Dubey 2012**	US	North America	O	Na	Bio mice	SC	Heart
15	**Esteves 2014**	Portugal	Europe	NS	Sero +	PCR	Nested	Brain/Diaphragm
16	**Fortier 1990**	Portugal	Europe	C	NA	Bio mice	IP	Brain/Heart/Diaphragm
17	**Frazao-Texeira 2006**	Brazil	South America	O	NA	Bio mice	IP	Brain
18	**Frazao-Texeira 2011**	Brazil	South America	NS	NA	Bio mice	Inoculation	Heart
19	**Frazao-Texeira 2011**	Brazil	South America	NS	NA	Bio mice	Inoculation	Brain
20	**Feitosa 2014**	Brazil	South America	NS	Sero +	Bio mice	SC	Brain/Heart/Muscle
21	**Gajadhar 1998**	Canada	North America	NS	NA	Bio mice	SC	Heart/Diapraghm
22	**Galvan-Ramirez 2010**	Mexico	Central America	NS	NA	Bio mice	SC	Muscle
23	**Gomez-Samblas 2015**	Spain	Europe	NS	NA	PCR	MC-PCR	Serrano ham
24	**Halova 2012**	Ireland	Europe	NS	NA	PCR	Nested	Diaphragm
25	**Jacobs 1960**	US	North America	NS	NA	Bio mice	IP	Diaphragm
26	**Jamra 1969**	Brazil	South America	NS	NA	Bio mice	IP	Muscle
27	**Jamra 1969**	Brazil	South America	NS	NA	Bio mice	IP	Sausages
28	**Martins 1989**	Brazil	South America	NS	NA	Bio mice	IP	Muscle
29	**Medonca 2004**	Brazil	South America	NS	NA	PCR	SC	Sausages
30	**Navarro 1992**	Brazil	South America	NS	NA	Bio mice	IP	Muscle
31	**Navarro 1992**	Brazil	North America	NS	NA	Bio mice	IP	Brain
32	**Rothe 1985**	Australia	Oceania	NS	NA	Bio mice	IP	Muscle
33	**Samico Fernandes 2012**	Brazil	North America	NS	NA	PCR	Nested	Heart
34	**Siam 1979**	Egypt	Africa	NS	NA	Bio mice	IP	Diaphragm/Muscle
35	**Siam 1979**	Egypt	Africa	NS	NA	Bio mice	IP	Sausages and Mortadella
36	**Sousa 2006**	Portugal	Europe	SF	Sero +	Bio mice	SC	Brain/Heart
37	**Turcekova 2013**	Slovakia	Europe	NS	Sero +	PCR	Nested	Brain/Heart
38	**Vostalova 2000**	Czech Republic	Europe	C	NA	Bio mice	IP	Brain/Diaphragm
39	**Wang 2012**	China	Asia	NS	NA	PCR	Real-T PCR	Muscle
40	**Wang 2013**	China	Asia	NS	NA	Bio mice	IP	Brain
41	**Warnekulasuriya 1998**	UK	Europe	NS	NA	PCR	Nested	Sausages dried/ cured

Bio mice = Bioassays in mice, IP = intra-peritoneal, SC = subcutaneous, MC-PCR = magnetic capture PCR, NS = not specified in the primary study, NA = Not Appliable, Sero+ = seropositive.

**Table 4:** General information about eligible studies reporting data for sheep.

ID	References	Country	Geographic area	Animal age	Analytical technique		Sampled organ
1	**Asgari 2011**	Iran	Asia	>12	PCR	Nested	Brain/Liver/Muscle
2	**Azizi 2014**	Iran	Asia	<12	PCR	Nested	Brain/Liver/Muscle
3	**Azizi 2014**	Iran	Asia	<12	PCR	Nested	Brain/Liver/Muscle
4	**Berger Scoch 2011(1)**	Switzerland	Europe	<12	PCR	Real Time PCR	Diaphragm
5	**Berger Scoch 2011(2)**	Switzerland	Europe	>12	PCR	Real Time PCR	Diaphragm
6	**Belbacha 2004**	Morocco	Africa	NS	Bio mice	Feed/IP	Brain
7	Boughattas 2013*	Tunisia	Africa	>12	PCR	PCR	Heart
8	da Silva 2009*	Brazil	South America	NS	Bio mice	NS	Heart/Diaphragm
9	Dubey 2008*	US	North America	<12	Bio mice	Feed	Heart
10	Dumetre 2006*	France	Europe	>12	Bio mice	IP	Heart
11	**Ergin 2009**	Turkey	Asia	NS	PCR	Nested	Brain/Muscle
12	**Ergin 2009**	Turkey	Asia	NS	PCR	Nested	Brain
13	**Gharbi 2013**	Tunisia	Africa	NS	PCR	Nested	Heart
14	Glor 2013*	Switzerland	Europe	NS	PCR	Real Time PCR	Brain/Muscle
15	**Halos 2010**	France	Europe	<12	Bio mice	IP	Heart
16	**Halos 2010**	France	Europe	>12	Bio mice	IP	Heart
17	**Halova 2012**	Ireland	Europe	NS	PCR	Nested	Diaphragm
18	**Jacobs 1960**	US	North America	NS	Bio mice	IP	Diaphragm
19	**Jacobs 1963**	New Zealand	Oceania	>12	Bio mice	IP	Brain/Diaphragm/Muscle
20	**Jamra 1969**	Brazil	South America	>12	Bio mice	IP	Muscle
21	**Khayeche 2013**	Tunisia	Africa	>12	PCR	Nested	Heart
22	**Maciel 2014**	Brazil	South America	NS	PCR	Nested	Brain
23	**Opsteegh 2010**	The Netherlands	Europe	NS	PCR	MC-PCR	Heart
24	Ragozo 2008*	Brazil	South America	NS	Bio mice	NS	Heart/Brain/Diaphragm
25	**Rahdar 2012**	Iran	Asia	<12	PCR	PCR	Tongue/Heart/Muscle
26	**Rothe 1985**	Australia	Oceania	<12	Bio mice	IP	Muscle
27	**Yildiz 2014**	Turkey	Asia	<12	Micro	NA	Brain/Diaphragm/Muscle
28	**Yildiz 2014**	Turkey	Asia	>12	Micro	NA	Brain/Diaphragm/Muscle
29	Vieira 2001*	Brazil	South America	NS	PCR	PCR	Brain/Diaphragm

Bio mice = Bioassays in mice, IP = intra-peritoneal, SC = subcutaneous, MC-PCR = magnetic capture PCR, NS = not specified in the primary study, NA = Not Appliable,

*studies that performed a serological screening.

### Cattle

The systematic review process identified 22 studies, presented in 18 papers, dealing with the direct identification of *T*. *gondii* in bovine meat [12,19,29–44]. However, one paper was not included in statistical analysis as it investigated only four samples [19]. General information about the 21 studies retrieved is presented in Table 2.

Meta-analysis, as summarized in S1 Fig, identified a pooled *T*. *gondii* prevalence of 2.6% (CI_95_ [0.5–5.8]). The 95% prediction interval ranged from 0% to 22%. Heterogeneity was high with significant Q test (p<0.0001), T^2^ = 0.0215 and I^2^ = 92% (details in Table 5).

**Table 5:** Summary of heterogeneity measures and Likelihood Ratio Test for each moderator tested in studies describing *T*. *gondii* prevalence in cattle.

	T^2^ (95%CI)	I^2^ (95%CI)	LRT p-value	R^2^
**No moderators**	0.0215 (0.0113–0.0620)	91.6 (85.3–96.9)	**-**	**-**
Geographic area*	0.0150 (0.0066–0.0589)	84.8 (71–95.6)	0.032	61.86
**Publication year**	0.0206 (0.0106–0.0613)	90.76 (83.48–96.69)	0.16	10.86
**Analytical technique**	0.0166 (0.0084–0.0601)	85.56 (74.91–95.54)	0.063	38.37
**Sample composition**	0.0232 (0.0121–0.0674)	91.33 (84.58–96.84)	0.89	0
**Sampling location**	0.0213 (0.0107–0.0626)	88.52 (79.52–95.78)	0.22	13.74

LRT = Likelihood Ratio Test

*statistically significant results.

**Fig 2:**
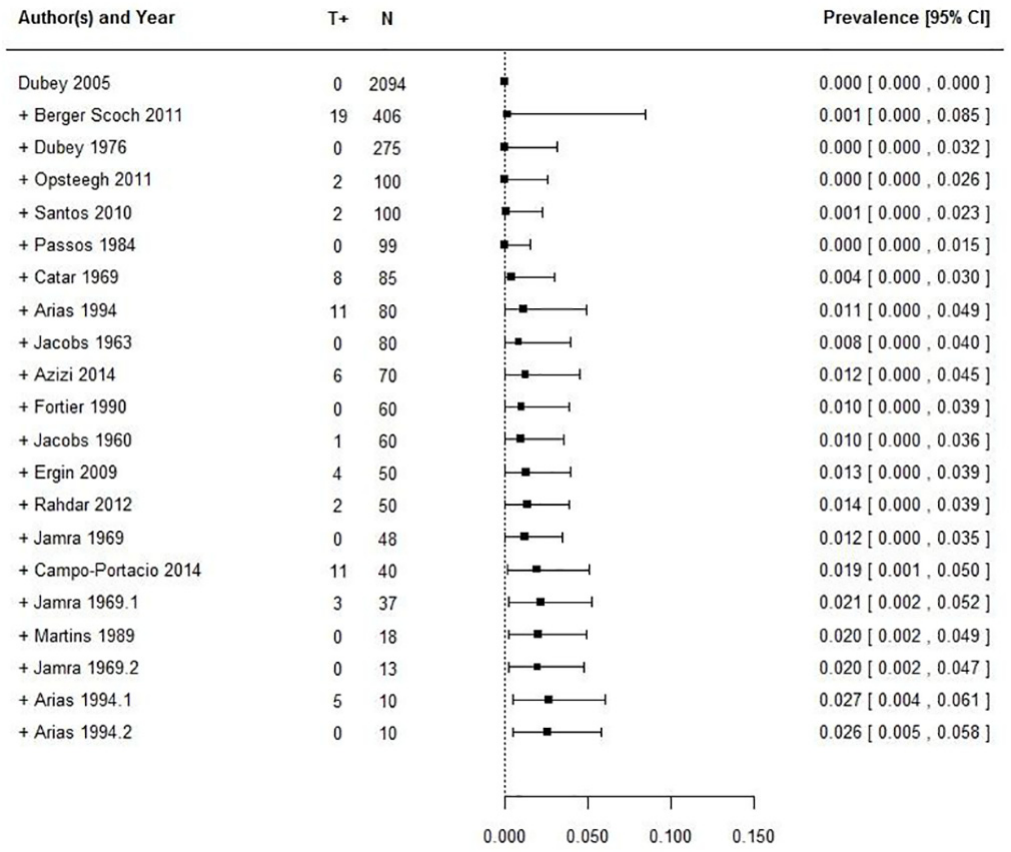
Cumulative meta-analysis on cattle studies based on decreasing sample size. T+ = positive samples, N = number of samples.

Sensitivity analysis identified study n°1 [29] and n°6 [32] as outliers according to externally studentized residuals, and their removal one by one resulted in a noticeable reduction of combined estimate, with a final pooled prevalence that would reach 1.9% in both cases. However, the other sensitivity indexes applied (DFFITS, the Cook’s distance, the hat function and the covariance ratio) did not identify these studies as influencing the final model according to *metafor* parameters. As regards publication bias, although the Trim and Fill test did not identify any asymmetry, a cumulative meta-analysis based on the number of samples (N) showed increasing prevalences as the number of samples in the studies decreased (Fig 2).

Because of the high level of heterogeneity observed, univariable meta-regressions were performed on publication year, geographic area, analytical technique, sample composition and sampling location. A significant effect was associated with geographic area, according to the Likelihood Ratio Test (p = 0.032), with a R^2^ of 61.9%. The multitest for pairwise comparison identified only one statistically significant difference (p = 0.0397), between Central America (K = 3 in one paper) and North America (K = 3 in three papers) prevalence estimates. The other moderators tested through meta-regression did not show any relevant impact according to the Likelihood Ratio Test (Table 5), suggesting that neither the analytical technique used, nor sample type or sampling location influenced *T*. *gondii* prevalence in a statistically significant way. Details of model coefficients and prevalence estimates are presented in Table 6.

**Table 6:** Summary of the output of univariable meta-regression in cattle or meat thereof for each category within moderators.

Moderator	K	N		Β	SE	Prevalence (95%CI)
**Epidemiological moderators**						
Geographic area*	3	170	Asia	0.2776	0.0806	0.060 (0.002–0.166)
	3	100	Central America	0.4263	0.0969	0.159 (0.040–0.328)
	4	655	Europe	0.1933	0.0661	0.022 (0.000–0.087)
	3	2429	North America	0.0592	0.0744	0.000 (0.000–0.026)
	1	80	Oceania	0.0557	0.1347	0.000 (0.000–0.085)
	7	355	South America	0.1988	0.0558	0.024 (0.000–0.078)
*Publication year*	21	3785		0.0027	0.0021	
**Methodological moderators**						
*Analytical technique*	2	2369	Bio cats	0.0203	0.0926	0.000 (0.000–0.025)
	12	600	Bio mice	0.19836	0.0447	0.024 (0.001–0.065)
	7	820	PCR	0.2672	0.0536	0.055 (0.012–0.0119)
*Sample composition*	14	3095	Single	0.2087	0.0460	0.032 (0.004–0.076)
	7	690	Pooled within	0.1973	0.0616	0.027 (0.000–0.087)
*Sampling location*	9	1315	Slaughterhouse	0.1499	0.0490	0.011 (0.000–0.048)
	9	2332	Retail	0.2792	0.0611	0.065 (0.014–0.142)
	3	138	NS	0.2256	0.0966	0.039 (0.000–0.154)

N = number of samples, K = number of studies, SE = Standard error, Bio = Bioassay,

*statistically significant results.

### Pigs

The systematic review process identified 41 studies, presented in 36 papers, dealing with the direct identification of *T*. *gondii* in pigs and meat thereof (details in Table 3) [12,31,33,36,37,39,40,45–69]. A univariable meta-regression was performed considering, as moderators, publication year, geographic area, analytical technique, farming system, sample type and sampling location.

The meta-analytical model (S2 Fig), without moderators, identified a *T*. *gondii* prevalence of 12.3% (CI_95_[7.6–17.7]). The 95% prediction interval ranged from 0% to 55%. Heterogeneity was high with significant Q test (p<0.0001), T^2^ = 0.0534 and I^2^ = 98% (details in Table 7).

**Table 7:** Summary of heterogeneity measures and Likelihood Ratio Test for each moderator tested in studies describing *T*. *gondii* prevalence in pigs.

	T^2^ (95%CI)	I^2^ (95%CI)	LRT p-value	R^2^
**No moderators**	0.0534 (0.0346–0.0931)	98.1 (97.1–98.9)	**-**	**-**
**Geographic area**	0.0499 (0.0313–0.0930)	97.8 (96.6–98.8)	0.17	22.79
Farming system*	0.0365 (0.0223–0.0629)	97.2 (95.5–98.4)	0.0004	37.31
**Publication year**	0.0545 0.0351 0.0952	98 (97–98.9)	0.52	0.65
**Analytical technique**	0.0524 (0.0335–0.0930)	97.4 (96–98.5)	0.24	7.86
**Sample composition**	0.0547 (0.0348–0.0971)	97.8 (96.6–98.7)	0.48	6.32
**Sampling location**	0.0550 (0.0353 0.0970)	98 (96.8–98.8)	0.58	2.64
**Serological screening**	0.0529 (0.0341–0.0934)	97.8 (96.6–98.7)	0.25	3.9

LRT = Likelihood Ratio Test

*statistically significant results.

Sensitivity analysis identified one study [70] as an outlier according to externally studentized residuals, but it was judged non influential according to *metafor* parameters. Its removal from the analysis resulted in a reduction of estimated prevalence up to 11.2%.

**Fig 3:**
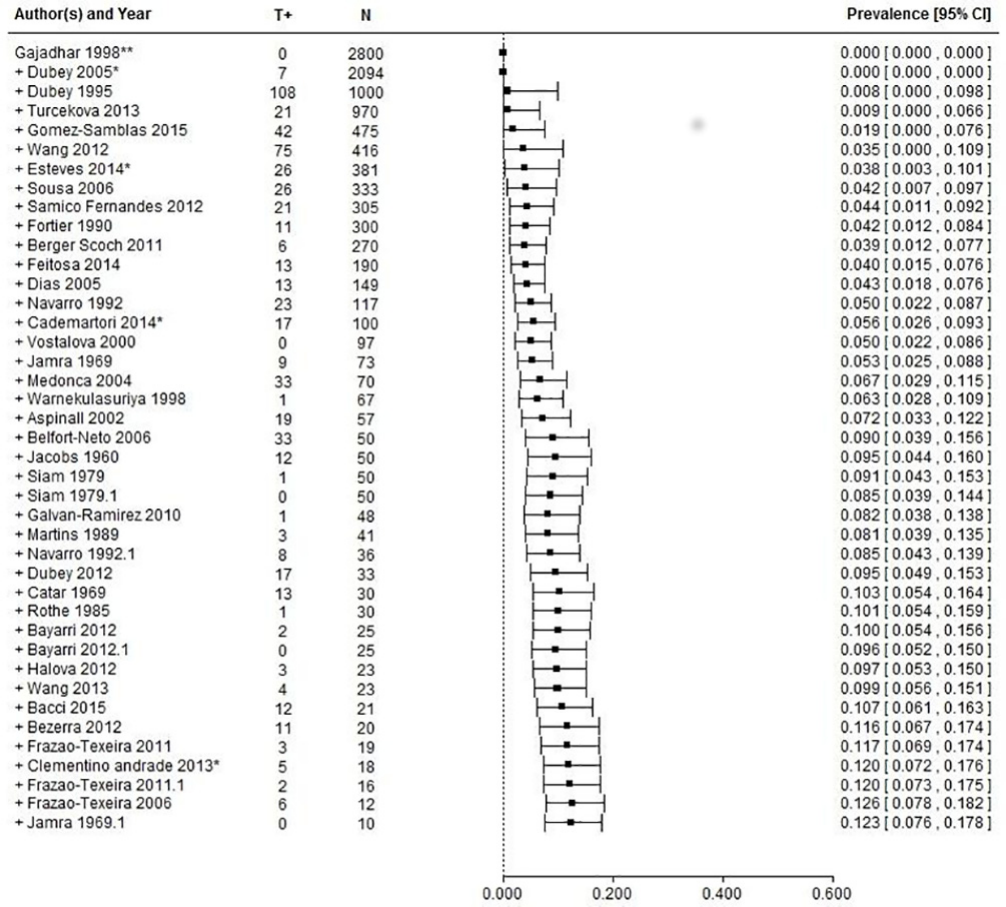
Cumulative meta-analysis on pig studies based on decreasing sample size. T+ = positive samples, N = number of samples.

A meta-regression based on publication year showed no significance. According to the Trim and Fill method, no asymmetry was identified. However, a cumulative meta-analysis based on the number of samples (N) showed increasing prevalences as the number of samples in the studies decreased (Fig 3).

Geographic area, analytical technique, sample type, sampling location and the presence of serological screening were not significant (p = 0.172, p = 0.239, p = 0.476, p = 0.576 and p = 0.25 respectively), and residual heterogeneity continued to be high according to T^2^ and I^2^ statistics (details in Table 7). Details of model coefficients and prevalence estimates are presented in Table 8. The only significant moderator was farming system (p = 0.0004) with R^2^ of 37.31, as organically farmed pigs had significantly higher *T*. *gondii* prevalences than pigs from conventional farms, small farms and from farms where this data was not reported (see Table 7).

**Table 8:** Summary of the output of univariable meta-regression analysis in pigs for each category within moderators.

Moderator	K	N		β	SE	Prevalence (95%CI)
**Epidemiological moderators**						
*Geographic area*	2	100	Africa	0.1201	0.1657	0.005 (0.000–0.178)
	2	493	Asia	0.4430	0.1660	0.176 (0.005–0.483)
	1	48	Central America	0.1734	0.2347	0.020 (0.000–0.347)
	14	3074	Europe	0.3159	0.0622	0.087 (0.027–0.172)
	7	6318	North America	0.3427	0.0868	0.104 (0.019–0.235)
	1	30	Oceania	0.2187	0.2411	0.037 (0.000–0.404)
	14	885	South America	0.4974	0.0643	0.221 (0.123–0.337)
Farming system*	2	397	Conventional	0.1253	0.1383	0.006 (0.000–0.141)
	4	86	Organic	0.8189	0.1106	0.534 (0.316–0.746)
	2	433	Small Farms	0.3558	0.1381	0.112 (0.000–0.140)
	33	9978	NS	0.3386	0.0355	0.101 (0.061–0.149)
*Publication year*	41	10894		0.0020	0.0026	
**Methodological moderators**						
*Analytical technique*	1	2094	Bio cats	0.0598	0.2292	0.000 (0.000–0.231)
	28	5695	Bio mice	0.3563	0.0459	0.113 (0.059–0.179)
	12	3105	PCR	0.4314	0.0681	0.167 (0.076–0.281)
*Sample composition*	21	4670	Single	0.4080	0.0541	0.149 (0.079–0.236)
	12	5321	Pooled within	0.3475	0.0694	0.107 (0.034–0.209)
	5	336	Meat products	0.3935	0.1109	0.139 (0.020–0.325)
	3	567	Cured meat products	0.1881	0.1404	0.025 (0.000–0.0193)
*Sampling location*	21	7094	Slaughterhouse	0.3649	0.0531	0.119 (0.057–0.197)
	18	3739	Retail	0.3604	0.0585	0.116 (0.049–0.202)
	2	61	NS	0.5496	0.1789	0.267 (0.029–0.616)
*Serological screening*	35	6808	No	0.3911	0.0417	0.137 (0.083–0.200)
	6	4086	Yes	0.2805	0.0889	0.067 (0.003–0.186)

K = number of studies, N = number of samples, SE = Standard error,

*statistically significant results.

### Sheep

The systematic review process identified 29 studies, presented in 24 papers, dealing with the direct identification of *T*. *gondii* in sheep meat [31,35,37–39,43,59,62,71–84]. General information about the 29 studies retrieved is presented in Table 9. Geographic area, analytical technique and animal age (coded in two categories) were included in the univariable meta-analysis as moderators.

**Table 9:** Summary of heterogeneity measures and Likelihood Ratio Test for each moderator tested in studies describing *Toxoplasma* prevalence in sheep.

	T^2^ (95%CI)	I^2^ (95%CI)	LRT p-value	R^2^
**No moderators**	0.0513 (0.0309–0.0988)	96.6 (94.4–98.2)	-	-
**Geographic area**	0.046 (0.0241–0.0887)	95.6 (92.5–97.8)	0.055	33.8
**Animal Age**	0.0470 (0.0279–0.0956)	96.1 (93.6–98.1)	0.13	15.9
**Publication year**	0.0532 (0.0319–0.1040)	96.7 (94.6–98.3)	0.88	0.2
**Analytical technique**	0.0470 (0.0278–0.0947)	96.2 (93.7–98.1)	0.11	15.9
Sample composition*	0.0458 (0.0269–0.0868)	96 (93.3–97.8)	0.03	14.1
**Sampling location**	0.0536 (0.0316–0.1044)	96.8 (94.7–98.3)	0.54	3.48
**Serological screening**	0.0529 (0.0316–0.1032)	96.6 (94.3–98.2)	0.61	1

LRT = Likelihood Ratio Test

*statistically significant results.

The meta-analytical model (S3 Fig), without moderators, identified a prevalence of 14.7% (CI_95_[8.9–21.5]. The 95% prediction interval ranged from 0% to 57%. Heterogeneity was high with significant Q test (p<0.0001), T^2^ = 0.0513 and I^2^ = 97% (details in Table 9).

Sensitivity analysis identified study n° 19 [38] as an outlier according to externally studentized residuals, but no influences in the model were highlighted according to other indexes investigated.

**Fig 4:**
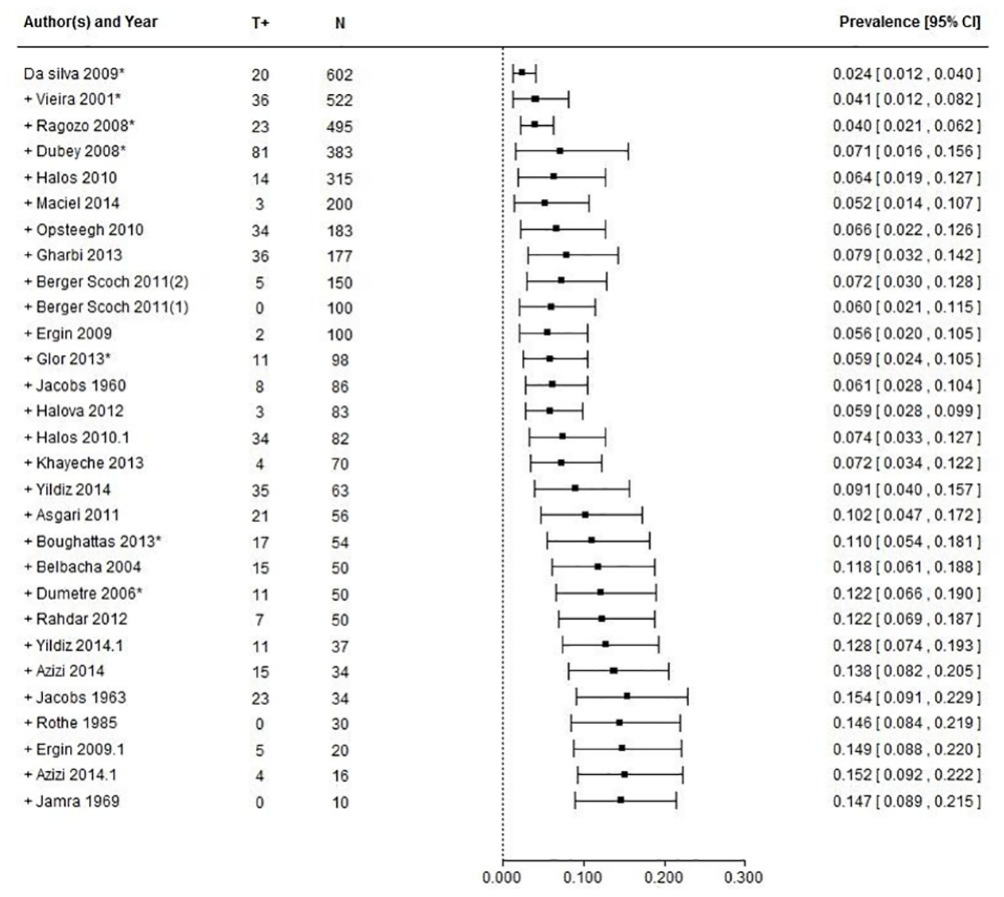
Cumulative meta-analysis on sheep studies based on decreasing sample size. T+ = positive samples, N = number of samples.

Cumulative meta-analysis based on publication year did not show any relevant trend. According to the Trim and Fill method, no asymmetry was identified. However, a cumulative meta-analysis based on the number of samples (N) showed increasing prevalences as the number of samples in the studies decreased (Fig 4).

None of the following moderators, studied using univariable meta-regression, were significant: geographic area (p = 0.0553), analytical technique (p = 0.1173), animal age (p = 0.1273), serological screening (p = 0.615), sampling location (p = 0.541), as summarized in Table 9. Sample composition was significant, with p = 0.031 and R^2^ value of 14.12%. Details of meta-regression coefficients and prevalence estimates are presented in Table 10.

**Table 10:** Summary of the output of univariable meta-regression analysis in sheep for each category within moderators.

Moderator	K	N		β	SE	Prevalence (95%CI)
**Epidemiological moderators**						
*Geographic area*	4	351	Africa	0.4752	0.1074	0.203 (0.059–0.399)
	8	376	Asia	0.5525	0.0786	0.271 (0.143–0.420)
	8	1061	Europe	0.3297	0.0750	0.096 (0.023–0.204)
	2	469	North America	0.4003	0.1489	0.144 (0.003–0.405)
	2	64	Oceania	0.5295	0.1586	0.250 (0.038–0.556)
	5	1829	South America	0.1946	0.0964	0.028 (0.000–0.132)
*Age*	8	991	<12 months	0.4125	0.0807	0.153 (0.054–0.287)
	9	543	>12 months	0.5171	0.0768	0.239 (0.120–0.381)
	12	2616	NS	0.3177	0.0643	0.089 (0.027–0.177)
*Publication year*	30	4150		0.0004	0.0031	
**Methodological moderators**						
*Analytical technique*	11	2137	Bio mice	0.4020	0.0685	0.146 (0.061–0.256)
	16	1913	PCR	0.3662	0.565	0.120 (0.055–0.204)
	2	100	Micro	0.7147	0.1616	0.428 (0.143–0.741)
Sample composition*	17	2123	Single	0.3287	0.0540	0.096 (0.039–0.170)
	12	2027	Pooled within	0.5116	0.0651	0.234 (0.133–0.353)
*Sampling location*	21	3686	Slaughterhouse	0.4289	0.0510	0.166 (0.096–0.249)
	7	414	Retail	0.3084	0.1004	0.083 (0.004–0.228)
	1	50	Slaughter/retail	0.2419	0.3934	0.139 (0.000–0.584)
*Serological screening*	22	1946	No	0.4163	0.0514	0.156 (0.088–0.239)
	7	2155	Yes	0.3666	0.0886	0.120 (0.027–0.259)

K = number of studies, N = number of samples, SE = Standard Error, Bio = Bioassays,

*statistically significant results.

### Multivariable meta-regression (cattle, pig, sheep)

Multivariable meta-regression was performed based on species, geographic origin of sampled animals and their interaction. Univariable analysis on the full dataset, using species as the moderator, resulted in a significant Likelihood Ratio Test (p = 0.0078). Moreover, the multitest pairwise comparison identified the estimate of *T*. *gondii* prevalence in cattle to be significantly lower than in sheep and pigs, whereas no differences were observed between these last two species. The addition of geographic area to this model gave no significant results, whereas the interaction between the two moderators was significant (p = 0.0212).

The multitest performed on the final model allowed the comparison of species prevalence within different geographic areas. In North America, Asia and Oceania, the *T*. *gondii* prevalences in sheep and pigs were significantly lower than the prevalence in cattle. In Europe and South America, *T*. *gondii* prevalences in sheep were significantly higher than in cattle but there was no difference in *T*. *gondii* prevalences in pigs and cattle.

### Goats and Horses

This systematic review process allowed the retrieval of the few studies available dealing with goats and horses, five and two studies, respectively.

Prevalence in goats is, according to literature, quiet heterogeneous. Samples of brain, tongue, liver, plus neck, intercostal, and femoral muscles from 22 goats from Shiraz abattoirs (Iran) were analysed in 2008. *T*. *gondii* was detected in five (23%) animals, and in at least one tissue, through nested PCR [85]. In East Brazil, mice bioassay demonstrated the presence of viable *T*. *gondii* in 1 out of 10 seropositive goats identified through ELISA anti IgG antibodies (DAT) among the total of 50 goats tested (prevalence 2.5%) [47]. A similar prevalence was described in the North East of Brazil after examination of tongues, brains and hearts from 102 goats at slaughter and positive results to nested PCR were found in 2.9%, 3.9% and 1% of these organs, respectively [86]. In North America, the hearts of 234 goats aged between 6 and 12 months and collected from local retail meat stores in Maryland were tested using Modified Agglutination Test (MAT) and 112 of them also using mice bioassay. *T*. *gondii* was isolated from 29 of 112 goats (26%) [87]. Finally, in China, liver, lung and lymph nodes from 403 Yunnan black goats were collected randomly from different administrative regions in Yunnan province, and B1 gene (a marker of *T*. *gondii*) was identified using PCR in 20 (5%) of the animals [88].

As regards horses, in a Brazilian study, Evers and colleagues in 2013 detected *T*. *gondii* in 14 out of 398 (3.5%) brain samples using bioassays in mice. The parasite was identified through PCR in two mice, but the others were found to be positive by IFAT (Indirect Fluorescent Antibody Test). All 398 horses were also tested by serology, and interestingly, 13 out of 14 horses positive by mouse bioassays tested negative by IFAT (<1:64). Moreover, only two bioassay positive horses tested positive by PCR [89]. In Egypt, meat and tissue samples from 150 horses were bioassayed in mice (pool of heart, liver, skeletal and diaphragmatic muscle) and 79 were positive (52.6%), with consequent isolation of the parasite from peritoneal fluid of inoculated animals [90].

## Discussion

This review describes the current knowledge about *T*. *gondii* prevalence in meat-producing animals in a systematic way. The results showed a pooled prevalence for cattle, pigs and sheep of, respectively, 2.6% (CI_95_[0.5–5.8]), 12.3% (CI_95_[6–17.7]), and 14.7% (CI_95_[8.9–21.5]). For goats and horses, the retrieved results can only provide some partial indications, but show that *T*. *gondii* infection is relevant in both species, and deserving of further attention.

Although pooling prevalence estimates originating from different animal species could be considered of limited value, it enabled us to statistically define *T*. *gondii* prevalence differences among them. Cattle are generally described as poor hosts for *T*. *gondii* and the role of this species is generally judged of limited importance in toxoplasmosis epidemiology [11]. Our results showed that prevalence estimates in cattle were usually lower than those of pigs and sheep. Interestingly, this lower prevalence was not observed in Europe and South America, highlighting the importance of geographic area in *T*. *gondii* prevalence estimation.

It should be noted that the pooled estimate of prevalence in cattle may suffer from some limitations that are likely to have caused an overestimation of mean prevalence. The first limitation is due to the problem of calculating variance for 0 prevalence estimates. The present work, in order to account for such results, applied the double arcsin transformation on event rates. This has the advantage of allowing this calculation without the use of continuity corrections that cause an underestimation of study weight [22]. The second limitation is due to a potential publication bias that was not detected by the Trim and Fill method but is suggested by the cumulative meta-analysis based on sample size. As an example, if we had considered only studies with sample sizes greater than 50, the pooled prevalence would have been 1% (CI_95_ [0.00–3.6]). This potential bias was not detected by the Trim and Fill method. This was probably because of the wide Confidence Interval in the final estimate that, starting from 0% in the case of cattle, also included the CI obtained after the exclusion of studies with n<50. However, this decreasing trend cannot be ignored. It can be supposed that an unknown number of small studies with results showing *T*. *gondii* prevalences of 0 would have failed to be published, or indeed, never started the publication procedure, thus supporting the theory of “winner’s curse” [91]. The inclusion of grey literature in the search strategies probably would have corrected this overestimation. However, the present review focused solely on papers published in peer-reviewed journals to enhance the methodological rigor of the current study and the conclusions drawn regarding prevalence. The low prevalence in cattle, confirmed by our estimates, together with the short persistence of viable *T*. *gondii* in bovine tissues [11] can be used to support the theory of limited bovine role in *T*. *gondii* epidemiology. However, despite these considerations, the role of beef in *T*. *gondii* human epidemiology cannot be easily ruled out, as this meat is eaten raw or undercooked in several countries with a consequent high probability of infection if *T*. *gondii* is present, as demonstrated elsewhere [13].

As regards the other meat-producing animal species, a lack of difference was observed between sheep and pigs in each investigated area. This lack of difference was partly due to the inclusion, within pigs, of studies reporting *T*. *gondii* prevalence in organically farmed pigs. Pooled estimates from pigs and sheep suffered problems, in term of publication bias, similar to that already observed for cattle. Therefore, in these cases too, the *T*. *gondii* prevalence could be overestimated.

The pooled prevalences obtained in the present work should be interpreted cautiously, due to the high level of heterogeneity observed. Nonetheless, they provide important clues regarding the ranking of different meat-producing animal species that is of critical importance in the context of food safety. This is because, currently, there are no measures in place at the slaughter level able to identify animals carrying *T*. *gondii* [92–94] and prevention is left to consumer behaviour.

Univariable meta-regression models were fitted to account for variables explaining the high level of heterogeneity observed. A summary of moderators included is available in Table 1. Geographic area was an important variable affecting *T*. *gondii* prevalence in cattle, as it accounted for 61.87% of observed heterogeneity. Its significance was probably due to different farming systems in countries from which the different studies originated. However, this significance was not seen in studies dealing with sheep and pigs. In the case of sheep, it could be supposed that the lack of a widespread intensive farming system determines a common level of exposure in different countries. In the case of pigs, the lack of significance of geographic area is difficult to explain, although it could simply be due to a high level of heterogeneity within the geographic areas examined.

Analytical technique was expected to be a relevant moderator. Bioassay in cats is considered to be the gold standard because of the high sensitivity of these definitive host animals to *T*. *gondii* infection, and because samples of high quantity can be fed to cats, maximizing the probability of parasite ingestion [51]. Moreover, diagnosis of infection is performed through oocyst recovery from faeces, with widely accepted techniques optimized due to their routine use in small animal clinics. Only three studies were found reporting the use of bioassays in cats to assess prevalence, two performed with cattle samples and one with pig samples. Moreover, these studies were performed by the same research group in the same geographic area, and thus, it is difficult to evaluate the significance of these different factors. An alternative, more frequently used, bioassay technique is performed using mice, an animal more familiar to researchers and research centres. However mice are not the definitive hosts of *T*. *gondii* and their sensitivity is considered to be lower [12]. This disadvantage is commonly addressed through the intra peritoneal or subcutaneous injection of the parasite to maximize the likelihood of infection. However, a major drawback is the consistent low quantity of sample analysed compared to the amount used in cat bioassay. PCR is often applied as an alternative solution but in this case too, the low quantity of sample analysed is a cause for concern. Moreover, PCR is unable to assess parasite viability, so consequently, overestimation of prevalence can occur. This weakness could be balanced out by the presence of false negatives due to the low amount of tissue from which DNA is extracted. The meta-regression applied in the current study failed to detect such differences in the meat-producing animal species investigated, due to the wide confidence interval produced, within different species, by each technique. Despite the Likelihood Ratio Test never being significant, pairwise comparison with p = 0.063 was found in the comparison between PCR and bioassays in cats within studies dealing with cattle. It is worth mentioning, in this case, that studies using cat bioassay always reported a 0 prevalence. However, due to the low number of such studies, statistical analysis was unable to define the estimate as significantly different from other techniques. PCR was not shown to overestimate prevalence in a statistically significant way, but was shown to result in a large confidence interval among different studies. PCR can be considered as a useful and more ethical method than bioassays to assess *T*. *gondii* prevalence in meat, but needs to be improved. In this context, methods able to concentrate DNA from larger quantities of samples through innovative techniques should be preferred [81].

Animal age is considered an important factor, as higher seropositivity is usually found in older animals [95], because the probability of an animal having had contact with the parasite increases with age. Papers retrieved in the current study rarely reported the age of tested animals and the use of this variable was possible only in the case of sheep, where differences between young (<12 months) and old (>12 months) animals were not observed.

Another important factor is farming system, as increased biosecurity level is able to minimize the contact of farmed animals with wildlife, cats and other potential sources of *T*. *gondii*. This information would be very interesting to rank as a relevant risk factor for *T*. *gondii* in animals/meat. Unfortunately, farming systems were rarely reported in the analysed studies, whereas these data are more common in studies dealing with seroprevalence as summarized elsewhere [15]. In the present work, only the organic farming system for pigs was able to be examined, and this system resulted in a significantly higher *T*. *gondii* prevalence rate in pigs/pork compared with pigs/pork from conventional pig farming. This result confirms published evidence, obtained through serological studies [96], and extends it, confirming *T*. *gondii* was significantly more prevalent in pork from organic farms than from conventional meat.

Other moderators classified as methodological were tested. Studies included in the present review considered different types of matrix and sample composition. We feel that examination of analysed organ as a moderator would have been very interesting. However, this was not possible because, often, studies reported that tissues from different organs were pooled within animals. Where analyses of individual organs were reported, results could not be defined as independent, impairing both subgroup analysis and moderator analysis.

Sample type was used as variable to differentiate samples composed of a single organ, samples composed of different organs from the same animal, meat products, assumed to be composed of meat from different animals, and cured meat products, assumed to be less contaminated. It is arguable that, following the uneven distribution of *T*. *gondii* within an animal [97], the pooling of different organs would increase the risk of positive findings. Moreover, meat products are considered to be of increased risk since they are, in effect, similar to a pooled sample [19]. Our analysis was not able to identify such differences according to a univariable meta-regression, in cattle and pigs. However, in sheep, samples pooled within the animal resulted in a significantly higher prevalence compared to single organ samples.

As expected, sampling location was found to be unrelated to prevalence because *T*. *gondii* exists along the meat chain, from freshly-slaughtered animals to meat and meat products. Finally the use of serological screening to detect seropositive samples, which were then further analysed through direct methods were applied in some studies within the pigs and sheep categories. There were no significant differences in *T*. *gondii* prevalences between studies where serological screening was used and studies where it was not used.

Goats are commonly considered as a species at high risk of *T*. *gondii* contamination and this has been confirmed by the collected data. However, the evidence is not strong and further analyses are needed. This is true in the case of horses too, as only two studies were retrieved, due, probably, to the fact that horse consumption is a local phenomenon. However the first isolation of *T*. *gondii* in this species dates back to 1979 [98] and horse meat is sometimes consumed raw or undercooked. Therefore, the role of this meat-producing animal species in the spread of *T*. *gondii* should no longer be overlooked.

## Conclusions

The results of this systematic review show that *T*. *gondii* prevalence in meat animals worldwide is not negligible and that direct detection of this parasite in meat presents a heterogeneous situation. The relative prevalence of *T*. *gondii* in different meat-producing animal species varies worldwide, and no generalized assumption can be made regarding the role of these animals and meat thereof in the dissemination of the parasite to humans. This observation, together with differences in food habits suggests a high variability of human *T*. *gondii* infection worldwide. Further research should better evaluate and report the risk factors of the animal population in each study (and in each published paper), which would allow their proper evaluation. Furthermore, methodological and epidemiological sources of heterogeneity need to be clarified. In general, raw or undercooked meat from cattle, pigs, sheep, horses and goats is a potential source of *T*. *gondii* and should not be consumed by at-risk groups in the population. Control options should be studied to lower *T*. *gondii* impact on the human population.

## Supporting Information

S1 Fig Forest plot showing the estimated prevalence (with 95% CI) of *Toxoplasma* in cattle for each study. In addition, results for each category (geographic origins) identified through univariable meta-regression are shown. T+ = positive samples, N = number of samples, RE = Random Effects.
(PDF)

S2 Fig Forest plot showing the estimated prevalence (with 95% CI) of *Toxoplasma* in pigs for each study. In addition, results for each category (farming system) identified through univariable meta-regression are shown. T+ = positive samples, N = number of samples, RE = Random Effects.
N = number of samples, C = Conventional farms, NS = studies not reporting farm features, O = Organic farms, SF = Small Farms, *studies applying serological screening before direction.
(PDF)

S3 Fig Forest plot showing the estimated prevalence (with 95% CI) of *Toxoplasma* in sheep for each study. In addition results for each category (Sample type) identified through univariable meta-regression are shown. T+ = positive samples, N = number of samples, RE = Random Effects.*studies applying serological screening before direction.
(PDF)

S1 Table PRISMA 2009 Checklist. (DOC)
