# Supplementary material for: Investigating the Determinants of Toxoplasma gondii Prevalence in Meat: A Systematic Review and Meta-Regression
Source: PLoS One. 2016 Apr 15;11(4):e0153856. doi: 10.1371/journal.pone.0153856 (PMC4833317; doi:10.1371/journal.pone.0153856)

S3 Fig. Forest plot showing the estimated prevalence (with 95% CI) of *Toxoplasma* in sheep for each study. In addition results for each category (Sample type) identified through univariable meta-regression are shown. T+=positive samples, N=number of samples, RE= Random Effects.*studies applying serological screening before direction.

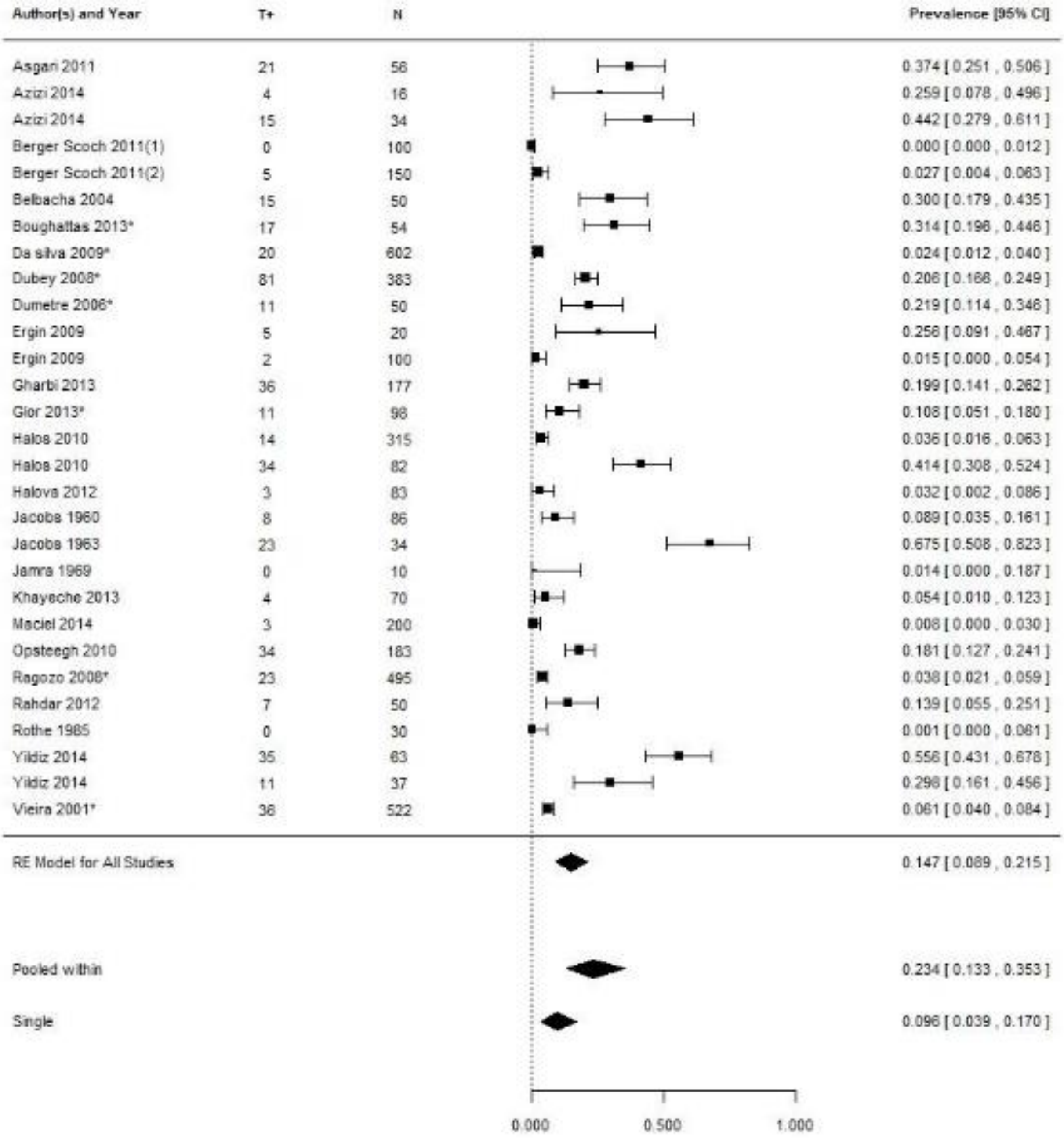

Supplement: S3 Fig — T+ = positive samples, N = number of samples, RE = Random Effects.*studies applying serological screening before direction. (PDF) [file pone.0153856.s003.pdf]
